# Differences in clinical features between current smokers and former smokers with OSA: a cross-sectional study

**DOI:** 10.1038/s41598-025-25032-1

**Published:** 2025-11-20

**Authors:** Zhifeng Chen, Yulin Shang, Binaya Wasti, Yanru Ou, Subo Gong, Xudong Xiang, Ruoyun Ouyang

**Affiliations:** 1 Department of Pulmonary and Critical Care Medicine, The Second Xiangya Hospital, Central South University, 139 Middle Renmin Road, Changsha, 410011 Hunan China; 2 Research Unit of Respiratory Disease, Central South University, 139 Middle Renmin Road, Changsha, 410011 Hunan China; 3 Clinical Medical Research Center for Pulmonary and Critical Care Medicine in Hunan Province, 139 Middle Renmin Road, Changsha, 410011 Hunan China; 4 Diagnosis and Treatment Center of Respiratory Disease in Hunan Province, 139 Middle Renmin Road, Changsha, 410011 Hunan China; 5 Ophthalmology and Otorhinolaryngology, Zigui County Hospital of Traditional Chinese Medicine, 30 Pinghu Avenue, Yichang, 443600 Hubei China; 6 B and C Medical College Teaching Hospital and Research Center, Mahendra Hwy, Birtamode, 57204 Nepal; 7 Department of Geriatrics, The Second Xiangya Hospital, Central South University, 139 Middle Renmin Road, Changsha, 410011 Hunan China; 8 Department of Emergency, The Second Xiangya Hospital, Central South University, 139 Middle Renmin Road, Changsha, 410011 Hunan China

**Keywords:** OSA, Current smokers, Former smokers, AHI, Smoking cessation, Respiratory tract diseases, Health occupations, Risk factors, Signs and symptoms

## Abstract

Smoking is both a cause of obstructive sleep apnea (OSA) and is an important reason for its rising prevalence. However, there is a lack of studies predicting smoking cessation specifically in patients with OSA. This study aimed to identify the factors linked to smoking cessation by examining and comparing the clinical characteristics of current smokers and former smokers with OSA. Eligible adults with a diagnosis of OSA and who were smokers (n = 504) were enrolled in the study. Data on demographics, PSG results, and clinical information were collected. Participants were categorized into current smokers and former smokers based on their smoking status. Logistic regression was used to analyze factors associated with smoking cessation and Cox proportional hazards regression to evaluate the interactions among these factors. Among all patients with OSA included in the study, 69.0% were current smokers, while 31.0% were former smokers. Compared to current smokers, former smokers were generally older, had a longer duration of OSA, exhibited a higher proportion of severe OSA, had more smoking pack-years and a longer smoking duration, a higher BMI, AHI, and ODI, and a lower MSaO_2_. Logistic regression analysis revealed that smoking cessation was positively associated with factors such as age, disease duration, AHI, BMI, various clinical manifestations, and comorbidities, but negatively associated with MSaO_2_. The Cox proportional hazards regression model indicated that among the factors related to smoking cessation, OSA severity interacted significantly with hyperuricemia, metabolic syndrome, obesity, and lacunar infarction (all *P* < 0.05). The factors related to smoking cessation identified in this study should be emphasized in interventions aimed at quitting smoking in OSA patients. Addressing these factors may help prevent the exacerbation of OSA and enhance patient outcomes.

## Introduction

Obstructive sleep apnea (OSA) is a respiratory syndrome caused by the collapse of the upper airway during sleep^1^. OSA is associated with a variety of diseases and complications, such as coronary heart disease, hypertension, diabetes, stroke, dyslipidemia, and cognitive impairment^2–4^. The clinical manifestations of OSA include drowsiness, snoring, waking up at night, apnea, reduced attention span and open mouth breathing, which often affect the life and work of OSA patients^5^. OSA has several risk factors, with smoking being a key contributor to both the development and worsening of the condition. Zeng et al. found a significant association between smoking and the occurrence of OSA^6^. Among OSA patients, compared with non-smokers, moderate to severe OSA is more common among smokers, and the AHI and oxygen saturation decline index are higher. Furthermore, the duration of smoking was significantly correlated with the severity of OSA^7^. Smoking can cause changes in sleep structure. Jaehne et al. reported that compared with non-smokers, smokers have less sleep time, longer sleep latency, more rapid eye movement sleep, and increased sleep apnea and leg movement^8^. Smoking can damage the neuromuscular protective reflexes of the upper airway. In animal models, smoke exposure led to a greater decrease in the respiratory rate due to laryngeal irritation and more apnea. So far, no direct reports of damage to the upper airway neuromuscular reflexes during sleep after smoke exposure have been found in humans^9^. Smoking often induces inflammatory responses and causes upper respiratory tract inflammation, which may increase the risk of OSA. In patients with moderate and severe OSA, an increase in the thickness of the lamina propria of the uvula mucosa was found, and only in smokers did the histology of the uvula mucosa show significant changes^7^.

Smoking cessation is a key measure for the prevention or treatment of OSA. Chen et al. found that age, pack-years, and BMI were identified as factors promoting smoking cessation in patients with asthma^10^. Pataka et al. reported that quitting smoking can reduce apnea and hypopnea index (AHI) in OSA patients^11^. However, there is currently limited data on smoking cessation in OSA patients. In this study, we aimed to discover the clinical characteristics of current and former smokers with OSA in order to identify the key factors driving smoking cessation.

## Patients and methods

### Study participants and definitions

**Fig. 1:**
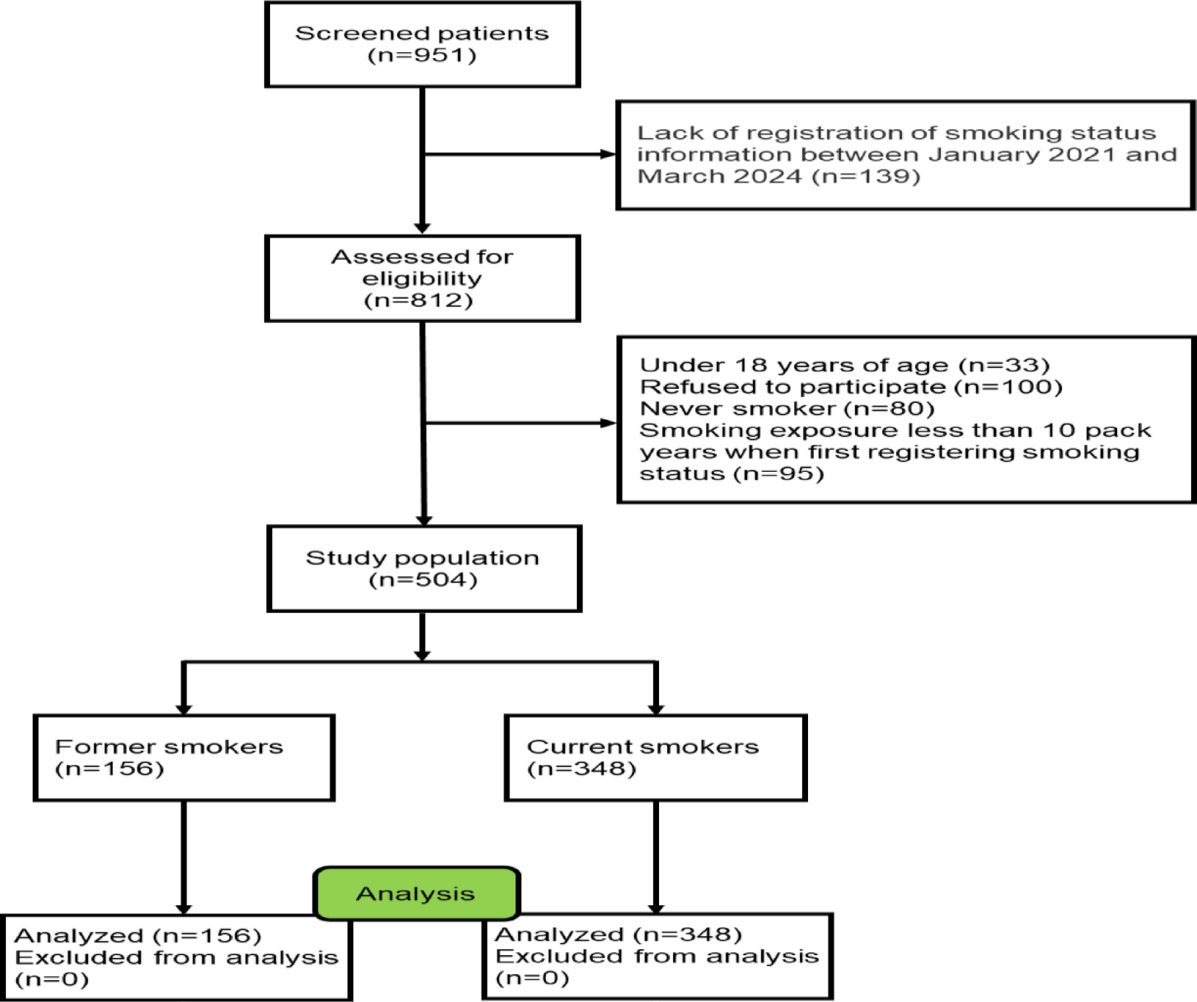
Flow diagram of the study.

This study was approved by the Ethics Review Committee of the Second Xiangya Hospital of Central South University (Ethical Code: LYF2023-059), all participants signed an informed consent form and all experiments were performed in accordance with the Declaration of Helsinki. Initially, we included 951 OSA patients diagnosed in the sleep laboratory at the Second Xiangya Hospital of Central South University (Hunan, China) between January 2021 and March 2024. OSA was diagnosed by polysomnography (PSG) according to the American Academy of Sleep Medicine Clinical Practice Guideline, with AHI ≥ 5 events/h^12^. The severity of OSA was defined by the AHI, with an AHI of 5–14 events/h for mild OSA, an AHI of 15–29 events/h for moderate OSA, and an AHI of ≥ 30 events/h for severe OSA^12^. All enrolled patients were at least 18 years of age. The non-smokers were defined as participants who had never smoked or had smoked fewer than 100 cigarettes in their lifetime. Smokers were defined as those who smoked continuously for more than 10 pack-years^10^. The former smokers were defined as participants who had quit smoking for at least six months prior to the study. Patients who had no history of smoking, were less than 10 pack-years, and were younger than 18 years old were excluded. A detailed description of the flow diagram for recruiting OSA patients can be found in Fig. 1.

### Data collection

After the written informed consent was collected, the subjects’ age, gender, duration of illness and smoking status were recorded. The duration of illness was defined as the time from the onset of OSA-related symptoms (such as snoring at night, apnea, daytime fatigue, drowsiness, etc.) in patients to the diagnosis of OSA in the hospital. Height and weight were measured and body mass index (BMI) was calculated. Meanwhile, the results of PSG tests (e.g., AHI, mean oxygen saturation [MSaO_2_], oxygen desaturation index [ODI]) were recorded.

### Patient selection

A total of 504 patients were included in this study (including 156 former smokers and 348 current smokers), and 447 patients were excluded (139 patients lacked registration records of smoking status, 100 patients who refused to participate in the study, 80 patients who never smoked, 95 patients who smoked less than 10 pack years, and 33 patients were younger than 18 years old).

### Statistical analysis

SPSS 26.0 software (IBM Corp.) was used for statistical analysis. Continuous variables were described as the mean and standard deviation (SD), and categorical variables were expressed as the number (percentage). The student’s t-test, Mann–Whitney U-test, and the chi-square test were used to determine the differences between the two groups. Univariate and multivariate logistic regression was used to calculate the odds ratio (OR) of each adjustment. Univariate and multivariate Cox proportional hazards regression was used to calculate each adjusted hazards ratio (HR). A *P* value < 0.05 indicated a statistically significant difference.

## Results

### Demographic characteristics

Table 1 shows the demographic and sociological characteristics of 504 participants, including 348 smokers and 156 former smokers. The mean age was 47.87 ± 11.78 years for current smokers and 56.43 ± 12.79 years for former smokers (Table 1). There were significant differences in age, OSA duration, severity of OSA, occupation, BMI, AHI, MSaO_2_, and ODI between current and former smokers. Compared to current smokers, former smokers were older, had longer OSA duration, more severe OSA, higher smoking pack-years, longer smoking duration, higher BMI, AHI, and ODI, and lower MSaO_2_. Among occupations, a higher percentage of clerks are current smokers compared to former smokers. Detailed information on participant characteristics is shown in Table 1.

**Table 1:** Demographic characteristics of the two groups.

Items	Current smokers (n = 348)	Former smokers (n = 156)	*P* value
Subjects, n (%)	348(69.0)	156(31.0)	
Demographics			
Age (y), M ± SD	47.87 ± 11.78	56.43 ± 12.79	< 0.001
< 45, n (%)	134(38.50)	28(17.90)	< 0.001
45–64	184(52.90)	84(53.80)	
≥ 65	30(8.60)	44(28.20)	
Sex M/F, n/n (%/%)	252/96(72.40)/(27.60)	116/40(74.40)/(25.60)	0.667
Disease duration (y)	6.61 ± 6.76	10.36 ± 7.73	< 0.001
Severity of OSA			< 0.001
Mild	190(54.60)	8(5.10)	
Moderate	125(35.90)	30(19.20)	
Severe	33(9.50)	118(75.60)	
Amount smoked, n (%)			
< 2 packs/day	326(93.70)	143(91.70)	0.450
≥ 2 packs/day	22(6.30)	13(8.30)	
Years of smoking (y)	19.04 ± 9.72	24.03 ± 11.88	< 0.001
Pack-years	24.57 ± 18.77	32.64 ± 24.48	< 0.001
Occupation, n (%)			0.003
Worker	49(14.10)	16(10.30)	
Farmer	38(10.90)	25(16.0)	
Clerk	232(66.70)	87(55.80)	
Retiree	29(8.30)	28(17.90)	
BMI (kg/m^2^)	27.19 ± 2.25	30.21 ± 2.85	< 0.001
Polysomnography indexes, M ± SD			
AHI (events/h)	19.74 ± 13.69	44.83 ± 18.36	< 0.001
MSaO_2_ (%)	92.58 ± 3.57	85.35 ± 5.54	< 0.001
ODI (events/h)	18.48 ± 12.10	43.84 ± 19.18	< 0.001

Comparisons were determined using Student’s t-test, the Mann–Whitney U-test, and the chi-square test between the two groups. *P* < 0.05 was considered statistically significant.

AHI, apnea and hypopnea; BMI, body mass index; MSaO_2_, mean oxygen saturation during sleep; ODI, oxygen desaturation index; OSA, obstructive sleep apnea.

### Clinical manifestations of the two groups

Table 2 shows the clinical manifestations of the two groups of patients. The incidence of hypomnesia, inattention, palpitations, gasping, hemoptysis, weakness, chest tightness, polydipsia, diuresis, and thirst was significantly higher in former smokers compared to current smokers (*P* < 0.05; Table 2). There was no significant difference in the incidence of other clinical symptoms between the two groups.

**Table 2:** Clinical manifestations of the two groups.

Items	Current smokers (n = 348)	Former smokers (n = 156)	*P* value
n (%)			
Drowsiness	173(49.70)	87(55.80)	0.212
Snore	158(45.40)	67(42.90)	0.629
Keep awake at night	140(40.20)	73(46.80)	0.173
Apnea	193(55.50)	79(50.60)	0.335
Open mouth breathing	190(54.60)	84(53.80)	0.923
Hypomnesia	22(6.30)	37(23.70)	< 0.001
Inattention	18(5.20)	29(18.60)	< 0.001
Palpitations	149(42.80)	68(43.60)	0.923
Swirl	184(52.90)	82(52.60)	1.000
Fever	125(35.90)	62(39.70)	0.426
Cough	111(31.90)	64(41.0)	0.054
Expectoration	91(26.10)	54(34.60)	0.056
Gasping	63(18.10)	54(34.60)	< 0.001
Dyspnea	9(2.60)	7(4.50)	0.278
Weakness	56(16.10)	73(46.80)	< 0.001
Chest distress	54(15.50)	76(48.70)	< 0.001
Headache	72(20.70)	92(59.0)	< 0.001
Polydipsia	67(19.30)	84(53.80)	< 0.001
Diuresis	36(10.30)	57(36.50)	< 0.001
Thirst	40(11.50)	69(44.20)	< 0.001

Comparisons were determined using the chi-square test between the two groups. *P* < 0.05 was considered statistically significant.

### Concomitant diseases of the two groups

Table 4 shows the concomitant diseases of the two groups. The incidence of chronic obstructive pulmonary disease (COPD), hypoxemia, asthma, bronchiectasis, hypertension, coronary heart disease, myocardial infarction, fatty liver, diabetes, hyperlipidemia, hyperuricemia, obesity, gout, metabolic syndrome, lacunar infarction, arteriosclerosis in lower extremities, carotid atherosclerosis, cerebral arteriosclerosis, and old cerebral infarction was higher in former smokers compared to current smokers. Additionally, the incidence of COPD and hypoxemia in former smokers was predominantly severe, whereas in current smokers it was mainly mild (*P* < 0.05; Table 3). There was no significant difference in the incidence of other complications between the two groups.

**Table 3:** Concomitant diseases of the two groups.

Items	Current smokers (n = 348)	Former smokers (n = 156)	*P* value
n (%)			
COPD			
Mild	34(9.80)	18(11.50)	< 0.001
Moderate	31(8.90)	30(19.20)	
Severe	25(7.20)	54(34.60)	
Hypoxemia			
Mild	41(11.80)	22(14.10)	< 0.001
Moderate	26(7.50)	42(26.90)	
Severe	23(6.60)	59(37.80)	
Pneumonia	69(19.80)	38(24.40)	0.289
Asthma	54 (15.50)	70 (44.90)	< 0.001
Nasosinusitis	59(17.0)	27(17.30)	0.899
Hypertension	78(22.40)	96(61.50)	< 0.001
Coronary heart disease	52(14.90)	80(51.30)	< 0.001
Myocardial infarction	26(7.50)	34(21.80)	< 0.001
Fatty liver	63(18.10)	77(49.40)	< 0.001
Chronic hepatitis B	38(10.90)	18(11.50)	0.878
Gallstone	46(13.20)	27(17.30)	0.273
Hepatic cyst	63(18.10)	35(22.40)	0.274
Chronic Gastritis	72(20.70)	29(18.60)	0.631
Gastric ulcer	52(14.90)	21(13.50)	0.784
Diabetes	88(25.30)	104(66.70)	< 0.001
Hyperlipidemia	82(23.60)	111(71.20)	< 0.001
Metabolic syndrome	82(23.60)	96(61.50)	< 0.001
Hyperuricemia	64(18.40)	85(54.50)	< 0.001
Obesity	118(33.90)	138(88.50)	< 0.001
Kidney stone	71(20.40)	34(21.80)	0.723
Gout	31(8.90)	43(27.60)	< 0.001
Benign Prostatic Hyperplasia	44(12.60)	17(10.90)	0.659
Cyst of kidney	56(16.10)	28(17.90)	0.607
Lacunar infarction	26 (7.50)	41(26.30)	< 0.001
Arteriosclerosis in the lower extremity	37(10.60)	50(32.10)	< 0.001
Carotid atherosclerosis	33(9.50)	40(25.60)	< 0.001
Cerebral arteriosclerosis	28(8.0)	53(34.0)	< 0.001
Old cerebral infarction	17(4.90)	46(29.50)	< 0.001

Comparisons were determined using the chi-square test between the two groups. *P* < 0.05 was considered statistically significant. COPD, chronic obstructive pulmonary disease.

### Univariate and multivariate logistic regression analysis of factors associated with smoking cessation based on sociodemographic and clinical manifestations

In view of the differences in clinical characteristics between former smokers and current smokers, univariate and multivariate logistic regression analysis was performed to identify factors related to smoking cessation. According to sociodemographic characteristics, univariate analysis identified age, disease duration, severity of OSA, smoking years, pack-years, BMI, AHI, ODI and lower MSaO_2_ as factors associated with smoking cessation. Multivariate analysis further revealed that patients who were older (OR = 1.096, 95% CI = 1.047–1.147, *P* < 0.001), had a longer duration of OSA (OR = 1.049, 95% CI = 1.000–1.100, *P* = 0.049), more severe OSA (OR = 66.310, 95% CI = 25.337–173.540, *P* < 0.001), higher BMI (OR = 1.897, 95% CI = 1.476–2.439, *P* < 0.001), higher AHI (OR = 90.830, 95% CI = 27.202–303.292, *P* < 0.001), and lower MSaO_2_ (OR = 0.730, 95% CI = 0.668–0.797, *P* < 0.001) were more likely to quit smoking.

Regarding clinical manifestations, univariate analysis identified hypomnesia, inattention, gasping, weakness, chest distress, headache, polydipsia, diuresis, and thirst as related to smoking cessation. Multivariate analysis further revealed that patients with hypomnesia (OR = 3.693, 95% CI = 1.274–10.701, *P* = 0.016), inattention (OR = 6.071, 95% CI = 1.955–18.850, *P* = 0.004), gasping (OR = 2.953, 95% CI = 1.323–6.589, *P* = 0.008), weakness (OR = 5.251, 95% CI = 1.725–15.984, *P* = 0.004), chest distress (OR = 4.239, 95% CI = 1.845–9.741, *P* = 0.001), polydipsia (OR = 3.566, 95% CI = 1.666–7.633, *P* = 0.009), diuresis (OR = 5.263, 95% CI = 2.086–13.280, *P* < 0.001), and thirst (OR = 5.736, 95% CI = 1.412–19.857, *P* = 0.015) were more likely to quit smoking (Table 4).

**Table 4:** Univariate and multivariate logistic regression analysis of factors associated with smoking cessation based on sociodemographic and clinical manifestations.

Variable	Univariate analysis		Multivariate analysis	
	*P* value	*OR* (95% CI)	*P* value	*OR* (95% CI)
Age	< 0.001	1.060 (1.042–1.079)	< 0.001	1.096 (1.047–1.147)
Disease duration	< 0.001	1.081 (1.052–1.110)	0.049	1.049 (1.000–1.100)
Severity of OSA				
Mild	Reference		Reference	
Moderate	< 0.001	5.70 (2.531–12.837)	< 0.001	6.186 (2.357–16.237)
Severe	< 0.001	84.924 (37.937–190.108)	< 0.001	66.310 (25.337–173.540)
Years of smoking	< 0.001	1.045 (1.026–1.065)		
Pack-years	< 0.001	1.018 (1.009–1.027)		
BMI	< 0.001	1.798 (1.587–2.038)	< 0.001	1.897 (1.476–2.439)
MSaO_2_	< 0.001	0.773 (0.739–0.808)	< 0.001	0.730 (0.668–0.797)
ODI	< 0.001	1.106 (1.086–1.126)		
Hypomnesia	0.001	4.607 (2.611–8.130)	0.016	3.693 (1.274–10.701)
Inattention	< 0.001	4.186 (2.246–7.703)	0.004	6.071 (1.955–18.850)
Gasping	< 0.001	2.395 (1.561–3.675)	0.008	2.953 (1.323–6.589)
Weakness	< 0.001	4.586 (2.998–7.015)	0.004	5.251 (1.725–15.984)
Chest distress	< 0.001	5.172 (3.373–7.931)	0.001	4.239 (1.845–9.741)
Headache	< 0.001	5.510 (3.653–8.313)		
Polydipsia	< 0.001	4.893 (3.239–7.391)	0.009	3.566 (1.666–7.633)
Diuresis	< 0.001	4.990 (3.104–8.020)	< 0.001	5.263 (2.086–13.280)
Thirst	< 0.001	6.107 (3.869–9.639)	0.015	5.736 (1.412–19.857)

Values were expressed as odds ratio (OR) and 95% confidence interval (CI). Factors associated with smoking cessation were determined by univariate and multivariate logistic regression analysis. Multivariate analysis was adjusted for age, sex, disease duration, occupation, BMI, pack-years and years of smoking. AHI, apnea and hypopnea index; BMI, body mass index; MSaO_2_, mean oxygen saturation during sleep; ODI, oxygen desaturation index; OSA, obstructive sleep apnea.

### Univariate and multivariate logistic regression analysis of factors associated with smoking cessation based on concomitant diseases

According to concomitant diseases, univariate analysis identified COPD, hypoxemia, asthma, hypertension, coronary heart disease, myocardial infarction, fatty liver, diabetes, hyperlipidemia, hyperuricemia, gout, metabolic syndrome, obesity, lacunar infarction, arteriosclerosis in the lower extremity, carotid atherosclerosis, cerebral arteriosclerosis, and old cerebral infarction as factors associated with smoking cessation.

Multivariate analysis further revealed that patients with more severe hypoxemia (OR = 21.961, 95% CI = 9.248–121.450, *P* = 0.040), asthma (OR = 3.373, 95% CI = 1.312–8.671, *P* = 0.012), hypertension (OR = 5.978, 95% CI = 2.543–14.054, *P* < 0.001), coronary heart disease (OR = 8.572, 95% CI = 3.377–21.758, *P* < 0.001), myocardial infarction (OR = 4.219, 95% CI = 1.286–13.835, *P* = 0.018), diabetes (OR = 6.708, 95% CI = 2.806–16.039, P < 0.001), hyperlipidemia (OR = 8.242, 95% CI = 3.433–19.790, *P* < 0.001), hyperuricemia (OR = 5.829, 95% CI = 2.386–14.238, *P* < 0.001), metabolic syndrome (OR = 3.304, 95% CI = 1.391–7.850, *P* < 0.001), and arteriosclerosis in the lower extremity (OR = 2.844, 95% CI = 1.050–7.706, *P* = 0.040) were more likely to quit smoking (Table 5).

**Table 5:** Univariate and multivariate logistic regression analysis of factors associated with smoking cessation based on concomitant diseases.

Variable	Univariate analysis		Multivariate analysis	
	*P* value	*OR* (95% CI)	*P* value	*OR* (95% CI)
COPD				
None	Reference			
Mild	0.004	2.518 (1.335–4.751)		
Moderate	< 0.001	4.624 (2.585–8.269)		
Severe	< 0.001	10.320 (5.910–18.022)		
Hypoxemia				
None	Reference		Reference	
Mild	< 0.001	4.569 (2.431–8.589)	0.586	5.587 (1.755–17.791)
Moderate	< 0.001	12.629 (6.871–23.213)	0.003	32.973 (9.859–110.274)
Severe	< 0.001	20.055 (10.975–36.647)	0.040	21.961 (9.248–121.450)
Asthma	< 0.001	4.432 (2.887–6.803)	0.012	3.373 (1.312–8.671)
Hypertension	< 0.001	5.538 (3.678–8.340)	< 0.001	5.978 (2.543–14.054)
Coronary heart disease	< 0.001	5.992 (3.895–9.217)	< 0.001	8.572 (3.377–21.758)
Myocardial infarction	< 0.001	3.451 (1.988–5.991)	0.018	4.219 (1.286–13.835)
Fatty liver	< 0.001	4.409 (2.909–6.683)		
Diabetes	< 0.001	5.909 (3.916–8.922)	< 0.001	6.708 (2.806–16.039)
Hyperlipidemia	< 0.001	8.002 (5.227–12.248)	< 0.001	8.242 (3.433–19.790)
Hyperuricemia	< 0.001	5.312 (3.505–8.051)	< 0.001	5.829 (2.386–14.238)
Gout	< 0.001	3.891 (2.338–6.475)		
Metabolic syndrome	< 0.001	5.190 (3.456–7.794)	< 0.001	3.304 (1.391–7.850)
Obesity	< 0.001	14.944 (8.717–25.617)		
Lacunar infarction	< 0.001	4.415 (2.585–7.543)		
Arteriosclerosis in the lower extremity	< 0.001	3.965 (2.456–6.400)	0.040	2.844 (1.050–7.706)
Carotid atherosclerosis	< 0.001	3.292 (1.981–5.469)		
Cerebral arteriosclerosis	< 0.001	5.881 (3.535–9.782)		
Old cerebral infarction	< 0.001	8.142 (4.483–14.787)		

Values were expressed as odds ratio (OR) and 95% confidence interval (CI). Factors associated with smoking cessation were determined by univariate and multivariate logistic regression analysis. Multivariate analysis was adjusted for age, sex, disease duration, occupation, BMI, pack-years and years of smoking. COPD, chronic obstructive pulmonary disease.

### Univariate and multivariate Cox proportional hazards regression analysis of the interaction between smoking cessation related factors

Consider that OSA is associated with comorbidities and there may be interactions. To further understand the effect of the interaction between OSA and comorbidities on smoking cessation, we assessed the interaction using univariate and multivariate Cox proportional hazards regression after adjusting for age, sex, occupation, BMI, pack-years and years of smoking. Univariate analysis showed that patients with severe OSA combined with severe hypoxemia, asthma, hypertension, coronary heart disease, myocardial infarction, diabetes, hyperlipidemia, hyperuricemia, and metabolic syndrome were significantly more likely to quit smoking than those with severe OSA alone.

Multivariate analysis further revealed that patients with severe OSA combined with hypertension (HR = 1.967, 95% CI = 1.316–2.940, *P* < 0.001), diabetes (HR = 2.383, 95% CI = 1.584–3.585, *P* < 0.001), hyperlipidemia (HR = 1.752, 95% CI = 1.070–2.870, *P* = 0.026), hyperuricemia (HR = 2.359, 95% CI = 1.564–3.557, *P* < 0.001), and metabolic syndrome (HR = 1.775, 95% CI = 1.141–2.761, *P* = 0.011) were more likely to quit smoking compared to patients with severe OSA alone (Table 6).

**Table 6:** Univariate and multivariate Cox proportional hazards regression analysis of the interaction between smoking cessation related factors.

Variable	Univariate analysis		Multivariate analysis	
Severe OSA &	*P* value	*HR* (95% CI)	*P* value	*HR* (95% CI)
Severe hypoxemia	< 0.001	3.191 (2.071–4.917)		
Asthma	< 0.001	2.120 (1.498–3.001)		
Hypertension	< 0.001	3.334 (2.384–4.664)	< 0.001	1.967 (1.316–2.940)
Coronary heart disease	< 0.001	2.906 (2.088–4.045)		
Myocardial infarction	< 0.001	2.196 (1.414–3.409)		
Diabetes	< 0.001	3.853 (2.741–5.416)	< 0.001	2.383 (1.584–3.585)
Hyperlipidemia	< 0.001	4.170 (2.935–5.925)	0.026	1.752 (1.070–2.870)
Hyperuricemia	< 0.001	3.895 (2.801–5.416)	< 0.001	2.359 (1.564–3.557)
Metabolic syndrome	< 0.001	3.949 (2.823–5.526)	0.011	1.775 (1.141–2.761)

Values were expressed as hazards ratio (HR) and 95% confidence interval (CI). The interaction of factors associated with smoking cessation was determined by univariate and multivariate Cox proportional hazards regression analysis. Multivariate analysis was adjusted for age, sex, occupation, BMI, pack-years and years of smoking.OSA, obstructive sleep apnea.

## Discussion

Smoking is a risk factor for several respiratory diseases, including obstructive sleep apnea. This cross-sectional descriptive study found differences between current and former smokers with OSA. Through logistic regression analysis, we identified independent factors that promote smoking cessation in OSA patients. Additionally, Cox proportional hazardss regression analysis revealed that OSA interacts with comorbidities, making patients more likely to quit smoking.

OSA was significantly associated with the risk of smokers compared to non-smokers^13^. Smoking behavior may contribute to the exacerbation of OSA by altering upper airway inflammation and causing sleep structure disorders^14^. Long uvula and enlarged tonsils are important causes of upper airway collapse of OSA. Kim et al. found that the lamina propria in uvula mucosa of OSA patients was significantly thickened in severe OSA. Meanwhile, compared with non-smokers, smokers increased the lamina propria thickness of uvula^7^. This study found that compared with current smokers, former smokers were older, had a longer OSA course, heavier OSA, more severe AHI, larger BMI and ODI, lower MSaO_2_, smoked longer, and had more pack-years of smoking. These findings suggest that most people with OSA continue to smoke until they develop obvious OSA symptoms themselves. Zeng et al. found an association between severe OSA, smoking years and smoking^6^. A cross-sectional study showed that in subjects with AHI > 50, smokers had a higher prevalence of OSA, and heavy smokers (≥ 30 pack-years) had a higher AHI than never smokers^15^. Casasola et al. showed that the amplitude and duration of the decrease of blood oxygen saturation during sleep in smokers was significantly higher than that in non-smokers, indicating that smoking caused more severe nighttime hypoxia^16^. In terms of occupation, it was found that the proportion of OSA patients who were clerks was higher, which may be related to the pressure of today’s society.

Pataka et al. found that quitting smoking can reduce AHI, which has guiding significance for the prognosis of OSA patients^11^. Long-term smoking cessation may be beneficial for improving sleep quality. Compared with those who have quit smoking, current smokers have much poorer sleep quality^17^. In addition, it has been found that the prevalence of sleep—disordered breathing among those who have quit smoking is not significantly higher than that among non-smokers^18^. Logistic regression analysis indicated that age, disease duration, OSA severity, AHI, ODI, BMI, and lower MSaO_2_ were factors associated with smoking cessation. Studies hava shown that old age and high BMI are strongly associated with OSA severity, which may encourage OSA patients to quit smoking^10,19^. OSA patients with high AHI are often accompanied by severe sleepiness, long disease duration, inattention, headache, and open mouth breathing, which affect the life and work of patients. Long-term smoking has been reported to increase the risk of airflow obstruction^20^. Studies have shown that the number of cigarettes smoked, the duration of smoking, and pack-years are important factors in quitting, and that the more cigarettes consumed and the longer people smoke, the more likely they are to quit^21–23^. Patients with a longer duration of disease may experience poorly controlled symptoms, which could motivate them to quit smoking. Chen et al. found that a longer disease duration increases the likelihood of smoking cessation^8^. Smoking cessation can lead to withdrawal symptoms, such as insomnia, increased wakefulness and sleep fragmentation, as well as reduced sleep time and sleep efficiency^24^. In addition, smoking cessation can also cause weight gain^25^. These factors can reduce the likelihood of successful smoking cessation and increase the relapse rate of smoking.

OSA is a risk factor for several diseases, such as cardiovascular disease, metabolic disease, cognitive impairment, and digestive disease^26–29^. In this study, in OSA patients, we found that compared with current smokers, former smokers had a higher incidence of respiratory diseases, cardiovascular diseases, digestive diseases, metabolic diseases, and neurological diseases, with corresponding increases in clinical manifestations. Naranjo et al. found that severe OSA could increase the hospitalization rate of COPD patients^30^. OSA causes abnormal blood pressure and metabolic disorders through hypoxia, endothelial cell dysfunction, inflammation and metabolic disorders, and promotes the occurrence of diseases^31^. Smoking can cause a series of physiological and pathological changes, such as oxidative stress and increased inflammation^32^. OSA disrupts sleep structure and causes repeated intermittent hypoxia during the night, leading to increased sympathetic nerve activity, heightened systemic inflammatory responses, and endothelial dysfunction^33^. Intermittent hypoxia also worsens metabolic dysfunction in obesity, contributing to increased insulin resistance and non-alcoholic fatty liver disease^34^. Additionally, oxidative stress associated with OSA promotes the proliferation of vascular smooth muscle, exacerbates inflammation, and accelerates arteriosclerosis^35^.

Studies have shown that quitting smoking is meaningful for preventing the occurrence and aggravation of many diseases^36^. Logistic regression analysis revealed that multiple comorbidities were associated with smoking cessation in patients with OSA, including hypoxemia, asthma, hypertension, coronary heart disease, myocardial infarction, diabetes, hyperuricemia, hyperlipidemia, metabolic syndrome, and arteriosclerosis of the lower extremities. Studies have shown that the incidence of cardiovascular disease is significantly reduced after smoking cessation^37^. Among OSA patients, smokers showed higher triglyceride levels and lower high-density lipoprotein levels compared to non-smokers^38^. Smoking cessation is an effective measure to reduce lung function decline, while also improving the sensitivity of patients to bronchodilators and inhaled corticosteroids^39^. Smoking cessation can increase insulin sensitivity and improve pancreatic β-cell function impairment in diabetic patients^40^. OSA causes damage to the hippocampus through lack of oxygen, leading to cognitive impairment^41^. Mons et al. found that smoking was associated with an increased risk of cognitive impairment, but that this risk decreased or even subsided after quitting^20^. A study found that smoking can promote an increase in uric acid. As tobacco exposure increases, the risk of hyperuricemia also rises. However, it is still unclear whether quitting smoking is beneficial for maintaining stable uric acid levels^42^. When OSA patients who smoke have these comorbidities, the probability of them quitting smoking may increase significantly.

Using Cox proportional hazardss regression, we found that OSA interacts with hypertension, diabetes, hyperlipidemia, hyperuricemia, and metabolic syndrome, and this interaction appears to facilitate smoking cessation in patients with OSA. Patients with OSA often have high blood pressure, hyperlipidemia, and a higher BMI than those without OSA^43^. The main features of OSA, namely intermittent hypoxia, can promote insulin resistance caused by increased adipose tissue inflammation, pancreatic beta cell dysfunction, hyperlipidemia, and decreased triglyceride clearance, resulting in aggravation of metabolic syndrome or increased risk of its occurrence^44^. Studies have shown that smokers’ smoking behavior can be influenced by health scares, reducing the likelihood of heavy smoking (> 20 cigarettes/day) and increasing the likelihood of smokers quitting, and OSA patients with these comorbidities may be motivated to quit smoking due to concerns about their own health^21^.

There were some limitations of this study: (a) This is a cross-sectional descriptive study. Therefore, the results of this study can only provide data related to smoking cessation, and we cannot draw conclusions about the direction of causation. (b) At present, there are few reported factors related to OSA and smoking cessation, so the mechanism needs to be further explored.

## Conclusions

In summary, among those who quit smoking, many quit smoking late and do not realize the need to quit until they have obvious clinical manifestations. The factors associated with smoking cessation identified in this study suggest that there are still clinical differences between current smokers and former smokers, and that these factors should be focused on in OSA cessation interventions or in people at high risk for OSA development to improve outcomes in OSA patients.

## Data Availability

The data used and analyzed in this study are available from the corresponding author on reasonable request; E-mail: ouyangruoyun@csu.edu.cn.
